# Supplementary material for: Disease Stage-Specific Pathogenicity of CD138 (Syndecan 1)-Expressing T Cells in Systemic Lupus Erythematosus
Source: Front Immunol. 2020 Jul 28;11:1569. doi: 10.3389/fimmu.2020.01569 (PMC7401833; doi:10.3389/fimmu.2020.01569)
Supplement: Supplementary file 1 [file Presentation_1.pptx]

## Slide 1
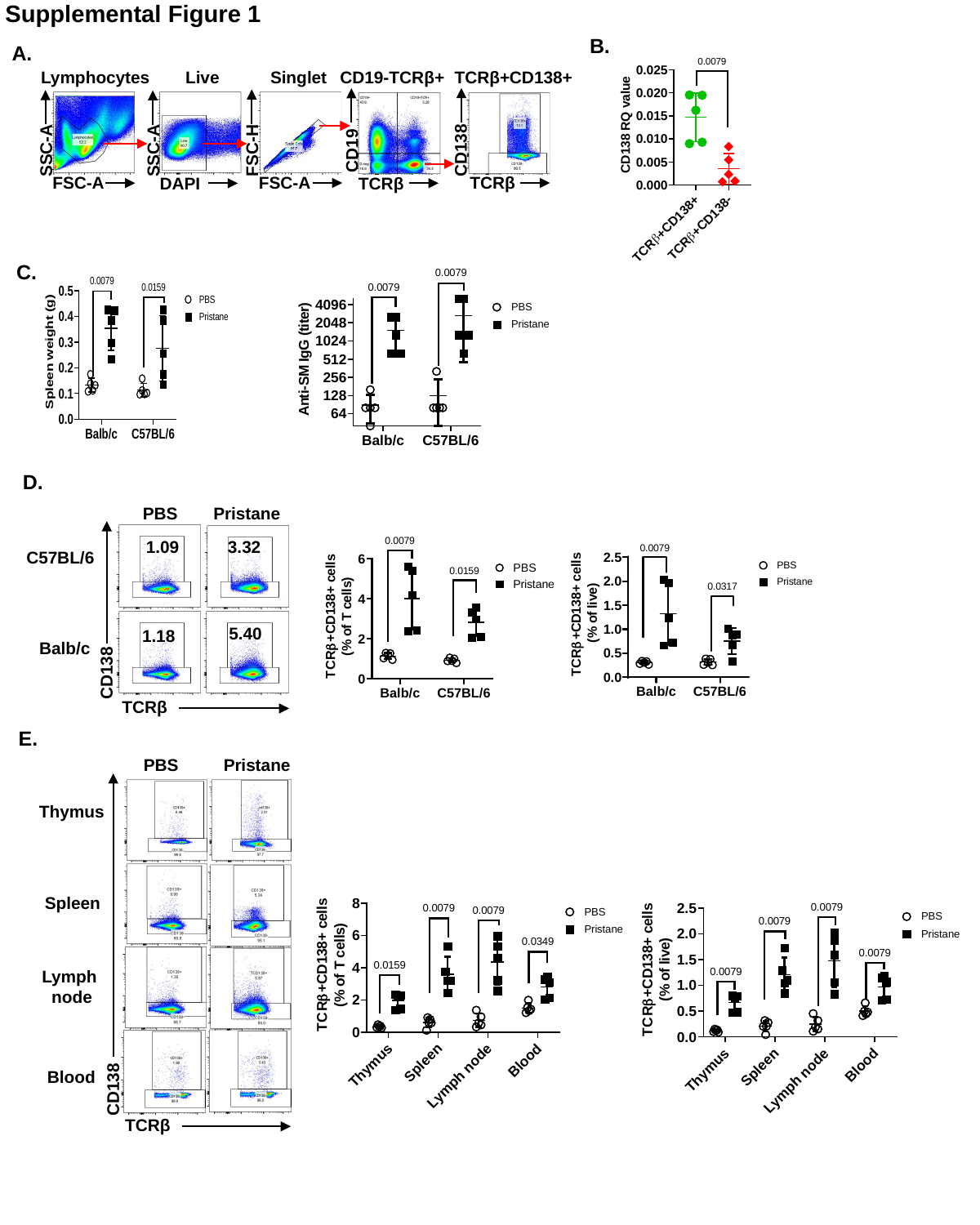

Supplemental Figure 1
B.
A.
Lymphocytes
Live
Singlet
CD19-TCRβ+
TCRβ+CD138+
FSC-H
CD138
SSC-A
CD19
SSC-A
FSC-A
TCRβ
FSC-A
TCRβ
DAPI
C.
D.
PBS
Pristane
1.09
3.32
C57BL/6
5.40
1.18
Balb/c
CD138
TCRβ
E.
PBS
Pristane
Thymus
Spleen
Lymph
node
Blood
CD138
TCRβ

## Slide 2
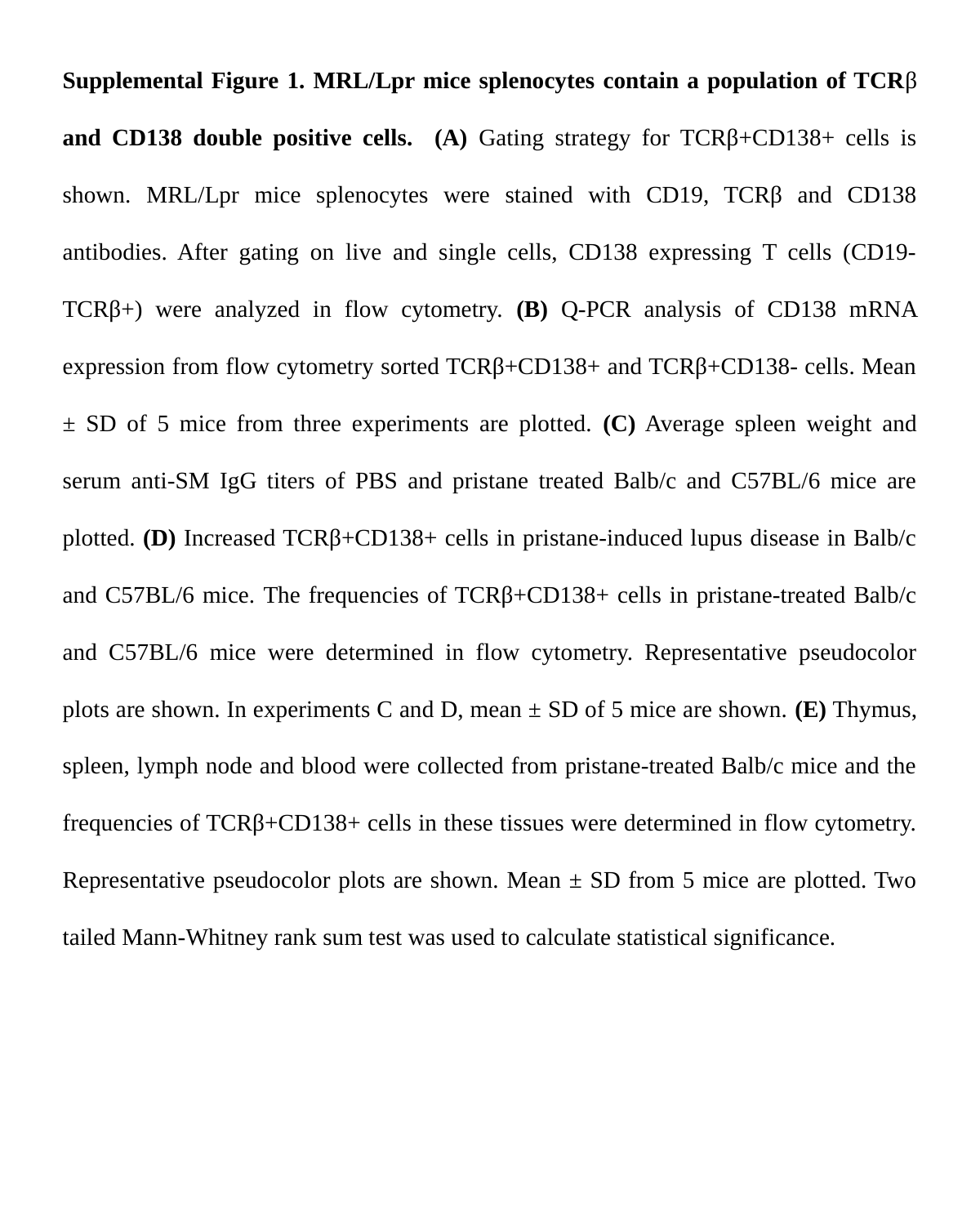

Supplemental Figure 1. MRL/Lpr mice splenocytes contain a population of TCRβ and CD138 double positive cells. (A) Gating strategy for TCRβ+CD138+ cells is shown. MRL/Lpr mice splenocytes were stained with CD19, TCRβ and CD138 antibodies. After gating on live and single cells, CD138 expressing T cells (CD19-TCRβ+) were analyzed in flow cytometry. (B) Q-PCR analysis of CD138 mRNA expression from flow cytometry sorted TCRβ+CD138+ and TCRβ+CD138- cells. Mean ± SD of 5 mice from three experiments are plotted. (C) Average spleen weight and serum anti-SM IgG titers of PBS and pristane treated Balb/c and C57BL/6 mice are plotted. (D) Increased TCRβ+CD138+ cells in pristane-induced lupus disease in Balb/c and C57BL/6 mice. The frequencies of TCRβ+CD138+ cells in pristane-treated Balb/c and C57BL/6 mice were determined in flow cytometry. Representative pseudocolor plots are shown. In experiments C and D, mean ± SD of 5 mice are shown. (E) Thymus, spleen, lymph node and blood were collected from pristane-treated Balb/c mice and the frequencies of TCRβ+CD138+ cells in these tissues were determined in flow cytometry. Representative pseudocolor plots are shown. Mean ± SD from 5 mice are plotted. Two tailed Mann-Whitney rank sum test was used to calculate statistical significance.

## Slide 3
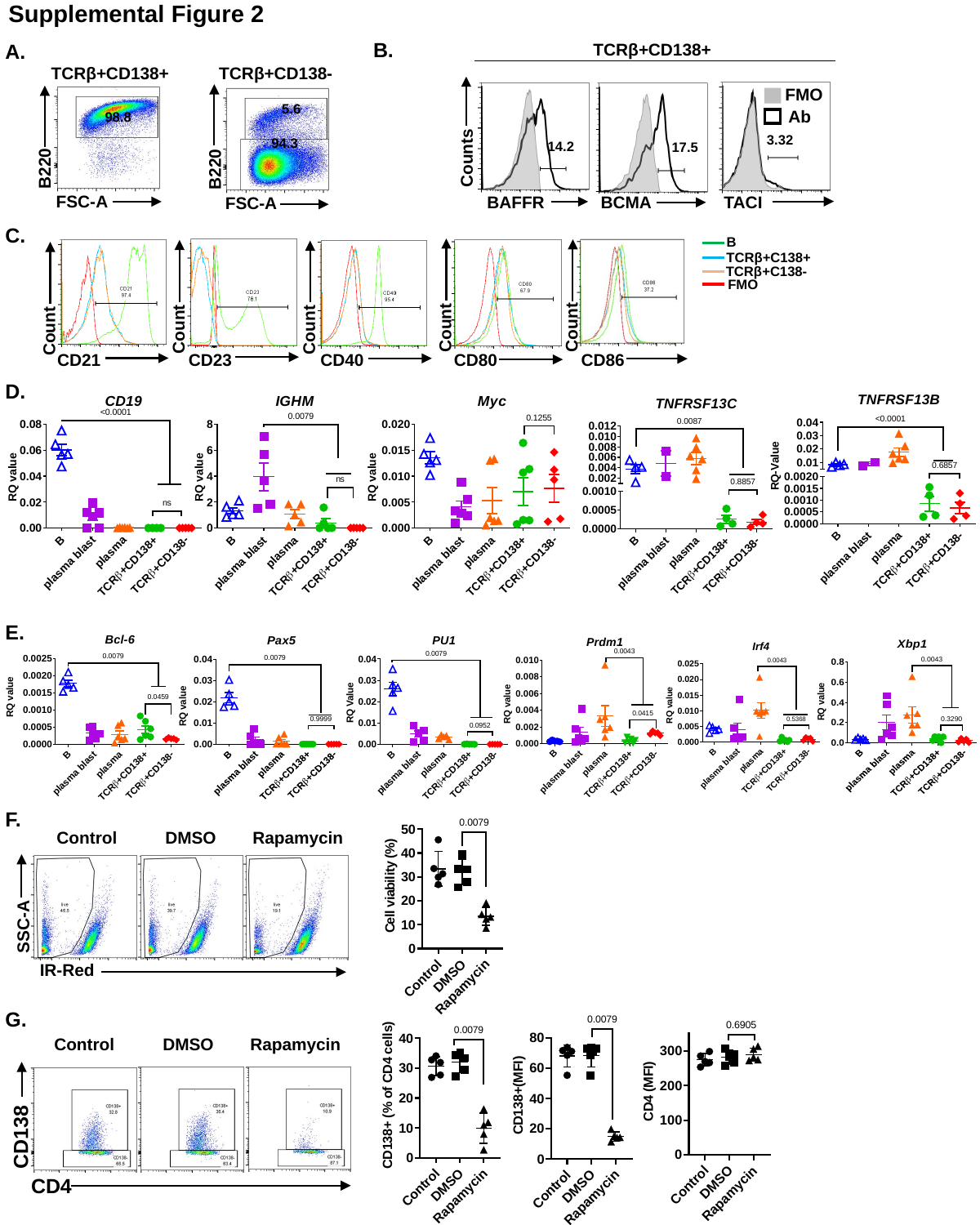

Supplemental Figure 2
B.
TCRβ+CD138+
A.
TCRβ+CD138+
TCRβ+CD138-
FMO
5.6
Ab
98.8
3.32
94.3
14.2
17.5
Counts
B220
B220
FSC-A
BAFFR
BCMA
TACI
FSC-A
C.
B
TCRβ+C138+
TCRβ+C138-
FMO
Count
Count
Count
Count
Count
CD21
CD23
CD40
CD80
CD86
D.
E.
F.
Control
DMSO
Rapamycin
SSC-A
IR-Red
G.
Control
DMSO
Rapamycin
CD138
CD4

## Slide 4
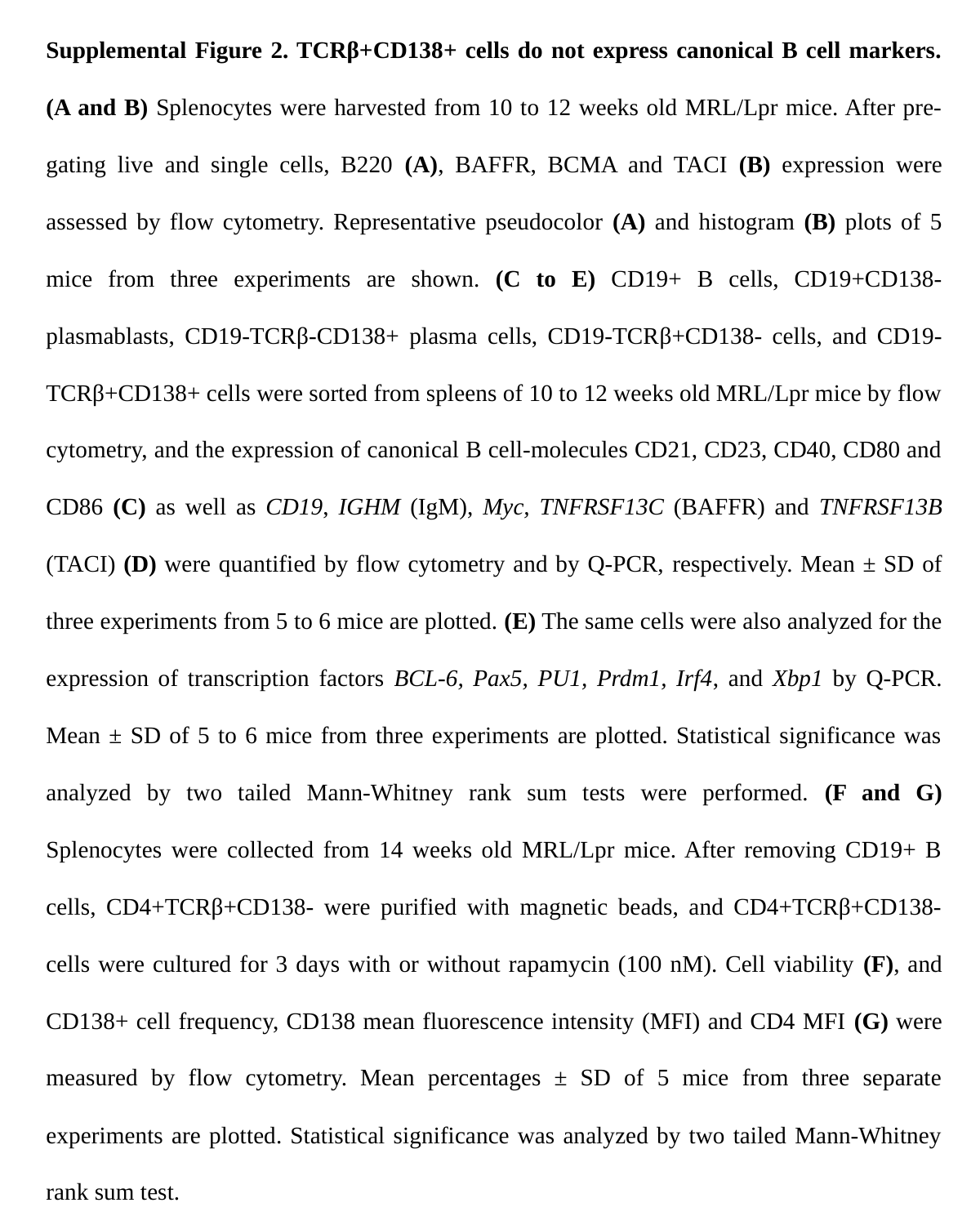

Supplemental Figure 2. TCRβ+CD138+ cells do not express canonical B cell markers. (A and B) Splenocytes were harvested from 10 to 12 weeks old MRL/Lpr mice. After pre-gating live and single cells, B220 (A), BAFFR, BCMA and TACI (B) expression were assessed by flow cytometry. Representative pseudocolor (A) and histogram (B) plots of 5 mice from three experiments are shown. (C to E) CD19+ B cells, CD19+CD138- plasmablasts, CD19-TCRβ-CD138+ plasma cells, CD19-TCRβ+CD138- cells, and CD19-TCRβ+CD138+ cells were sorted from spleens of 10 to 12 weeks old MRL/Lpr mice by flow cytometry, and the expression of canonical B cell-molecules CD21, CD23, CD40, CD80 and CD86 (C) as well as CD19, IGHM (IgM), Myc, TNFRSF13C (BAFFR) and TNFRSF13B (TACI) (D) were quantified by flow cytometry and by Q-PCR, respectively. Mean ± SD of three experiments from 5 to 6 mice are plotted. (E) The same cells were also analyzed for the expression of transcription factors BCL-6, Pax5, PU1, Prdm1, Irf4, and Xbp1 by Q-PCR. Mean ± SD of 5 to 6 mice from three experiments are plotted. Statistical significance was analyzed by two tailed Mann-Whitney rank sum tests were performed. (F and G) Splenocytes were collected from 14 weeks old MRL/Lpr mice. After removing CD19+ B cells, CD4+TCRβ+CD138- were purified with magnetic beads, and CD4+TCRβ+CD138- cells were cultured for 3 days with or without rapamycin (100 nM). Cell viability (F), and CD138+ cell frequency, CD138 mean fluorescence intensity (MFI) and CD4 MFI (G) were measured by flow cytometry. Mean percentages ± SD of 5 mice from three separate experiments are plotted. Statistical significance was analyzed by two tailed Mann-Whitney rank sum test.

## Slide 5
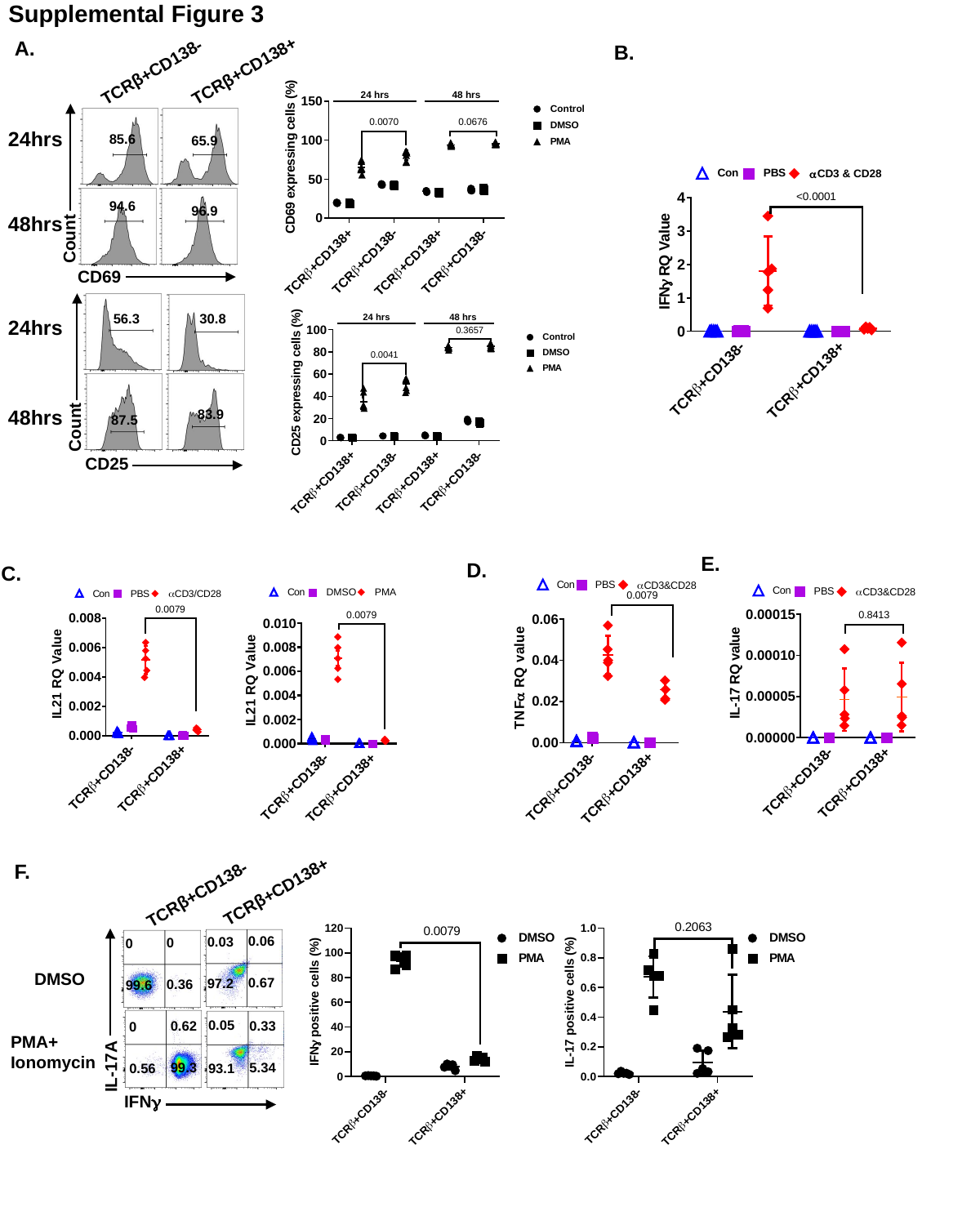

Supplemental Figure 3
A.
B.
TCRβ+CD138+
TCRβ+CD138-
24hrs
85.6
65.9
94.6
96.9
48hrs
Count
CD69
30.8
56.3
24hrs
48hrs
83.9
87.5
Count
CD25
E.
D.
C.
F.
TCRβ+CD138+
TCRβ+CD138-
0.06
0.03
0
0
DMSO
0.67
97.2
0.36
99.6
0.05
0.62
0.33
0
PMA+
Ionomycin
IL-17A
99.3
5.34
0.56
93.1
IFNγ

## Slide 6
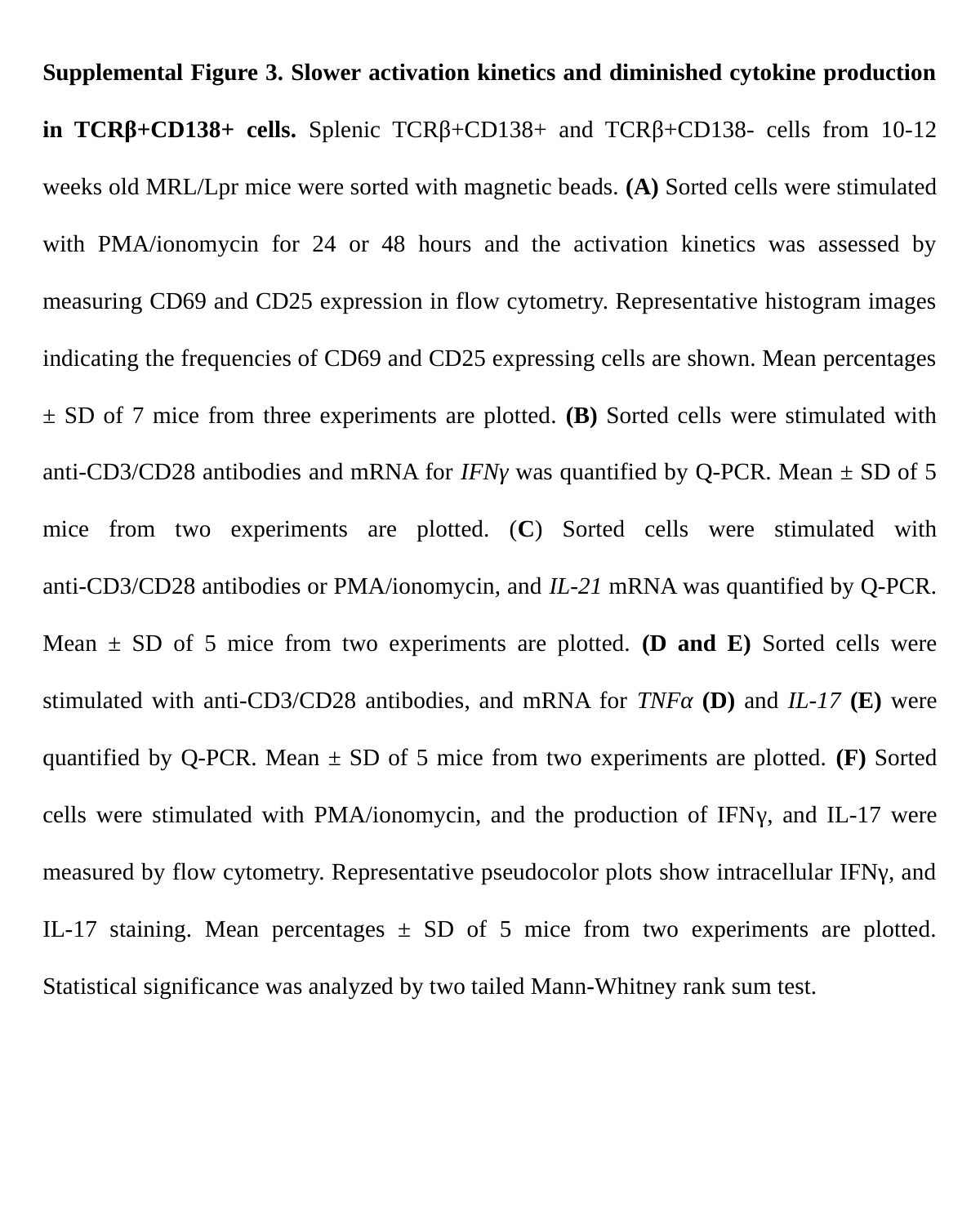

Supplemental Figure 3. Slower activation kinetics and diminished cytokine production in TCRβ+CD138+ cells. Splenic TCRβ+CD138+ and TCRβ+CD138- cells from 10-12 weeks old MRL/Lpr mice were sorted with magnetic beads. (A) Sorted cells were stimulated with PMA/ionomycin for 24 or 48 hours and the activation kinetics was assessed by measuring CD69 and CD25 expression in flow cytometry. Representative histogram images indicating the frequencies of CD69 and CD25 expressing cells are shown. Mean percentages ± SD of 7 mice from three experiments are plotted. (B) Sorted cells were stimulated with anti-CD3/CD28 antibodies and mRNA for IFNγ was quantified by Q-PCR. Mean ± SD of 5 mice from two experiments are plotted. (C) Sorted cells were stimulated with anti-CD3/CD28 antibodies or PMA/ionomycin, and IL-21 mRNA was quantified by Q-PCR. Mean ± SD of 5 mice from two experiments are plotted. (D and E) Sorted cells were stimulated with anti-CD3/CD28 antibodies, and mRNA for TNFα (D) and IL-17 (E) were quantified by Q-PCR. Mean ± SD of 5 mice from two experiments are plotted. (F) Sorted cells were stimulated with PMA/ionomycin, and the production of IFNγ, and IL-17 were measured by flow cytometry. Representative pseudocolor plots show intracellular IFNγ, and IL-17 staining. Mean percentages ± SD of 5 mice from two experiments are plotted. Statistical significance was analyzed by two tailed Mann-Whitney rank sum test.

## Slide 7
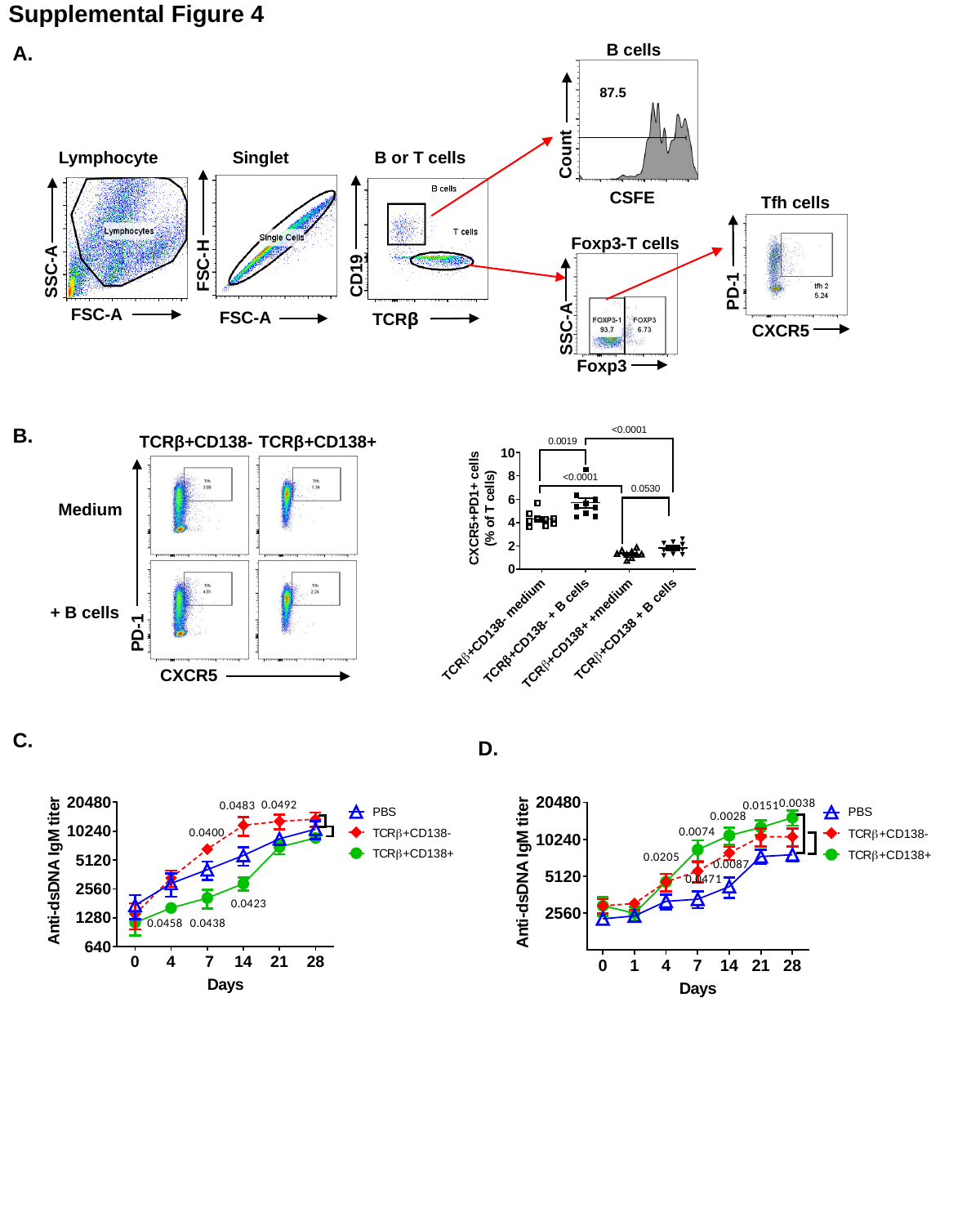

Supplemental Figure 4
B cells
A.
87.5
Count
Lymphocyte
Singlet
B or T cells
CSFE
Tfh cells
Foxp3-T cells
FSC-H
SSC-A
CD19
PD-1
FSC-A
FSC-A
TCRβ
SSC-A
CXCR5
Foxp3
B.
TCRβ+CD138+
TCRβ+CD138-
Medium
+ B cells
PD-1
CXCR5
C.
D.

## Slide 8
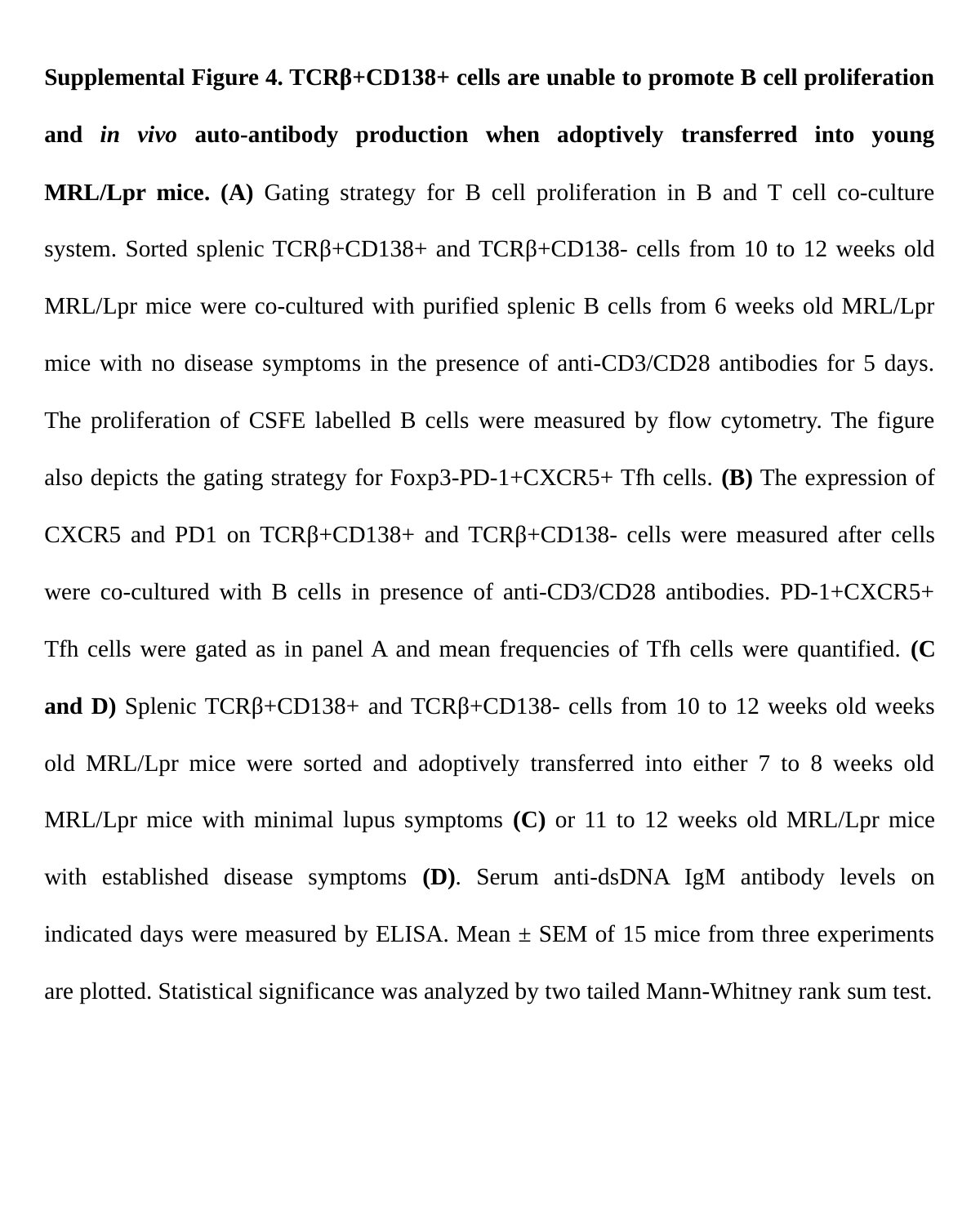

Supplemental Figure 4. TCRβ+CD138+ cells are unable to promote B cell proliferation and in vivo auto-antibody production when adoptively transferred into young MRL/Lpr mice. (A) Gating strategy for B cell proliferation in B and T cell co-culture system. Sorted splenic TCRβ+CD138+ and TCRβ+CD138- cells from 10 to 12 weeks old MRL/Lpr mice were co-cultured with purified splenic B cells from 6 weeks old MRL/Lpr mice with no disease symptoms in the presence of anti-CD3/CD28 antibodies for 5 days. The proliferation of CSFE labelled B cells were measured by flow cytometry. The figure also depicts the gating strategy for Foxp3-PD-1+CXCR5+ Tfh cells. (B) The expression of CXCR5 and PD1 on TCRβ+CD138+ and TCRβ+CD138- cells were measured after cells were co-cultured with B cells in presence of anti-CD3/CD28 antibodies. PD-1+CXCR5+ Tfh cells were gated as in panel A and mean frequencies of Tfh cells were quantified. (C and D) Splenic TCRβ+CD138+ and TCRβ+CD138- cells from 10 to 12 weeks old weeks old MRL/Lpr mice were sorted and adoptively transferred into either 7 to 8 weeks old MRL/Lpr mice with minimal lupus symptoms (C) or 11 to 12 weeks old MRL/Lpr mice with established disease symptoms (D). Serum anti-dsDNA IgM antibody levels on indicated days were measured by ELISA. Mean ± SEM of 15 mice from three experiments are plotted. Statistical significance was analyzed by two tailed Mann-Whitney rank sum test.

## Slide 9
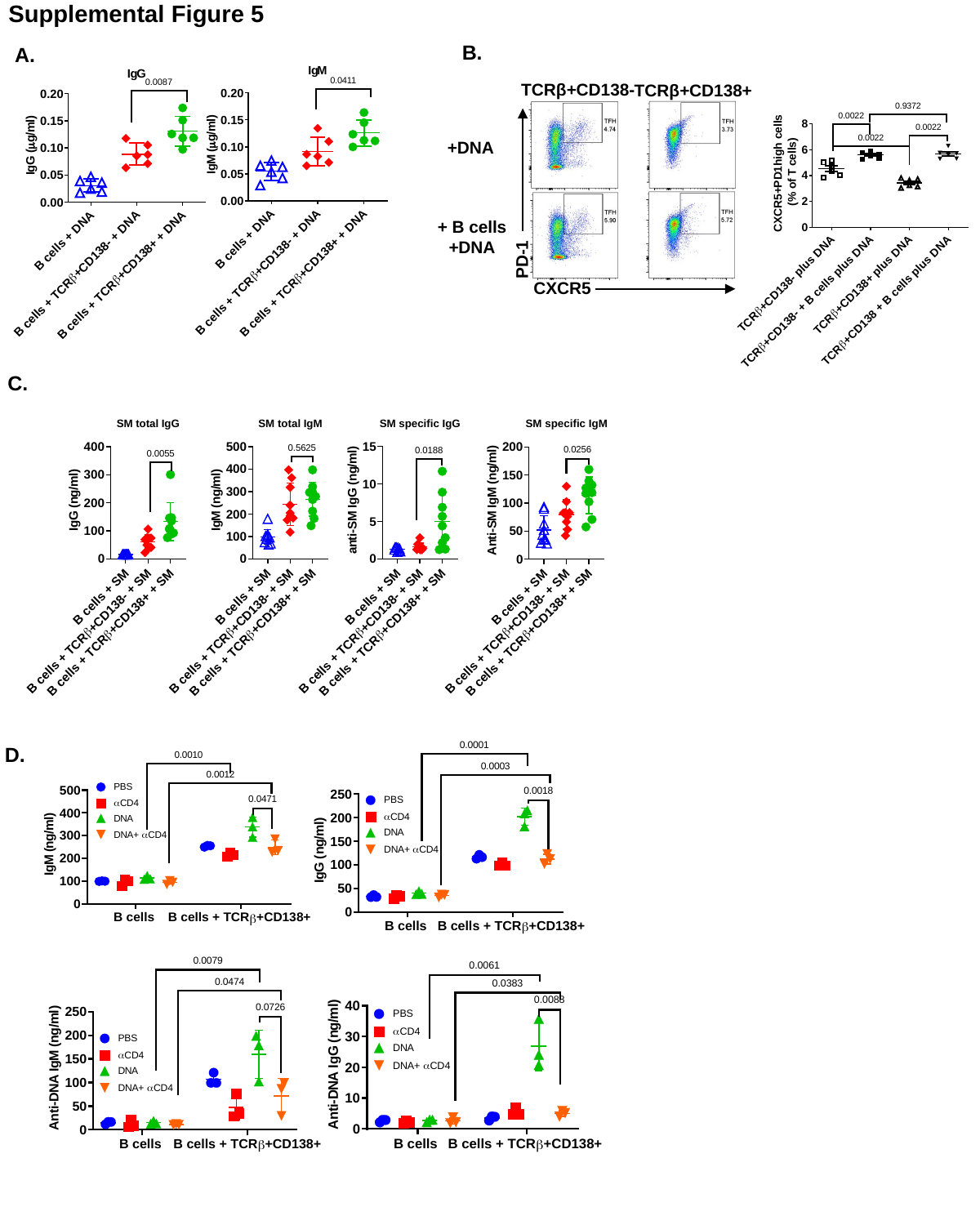

Supplemental Figure 5
B.
A.
TCRβ+CD138-
TCRβ+CD138+
+DNA
+ B cells
+DNA
PD-1
CXCR5
C.
D.

## Slide 10
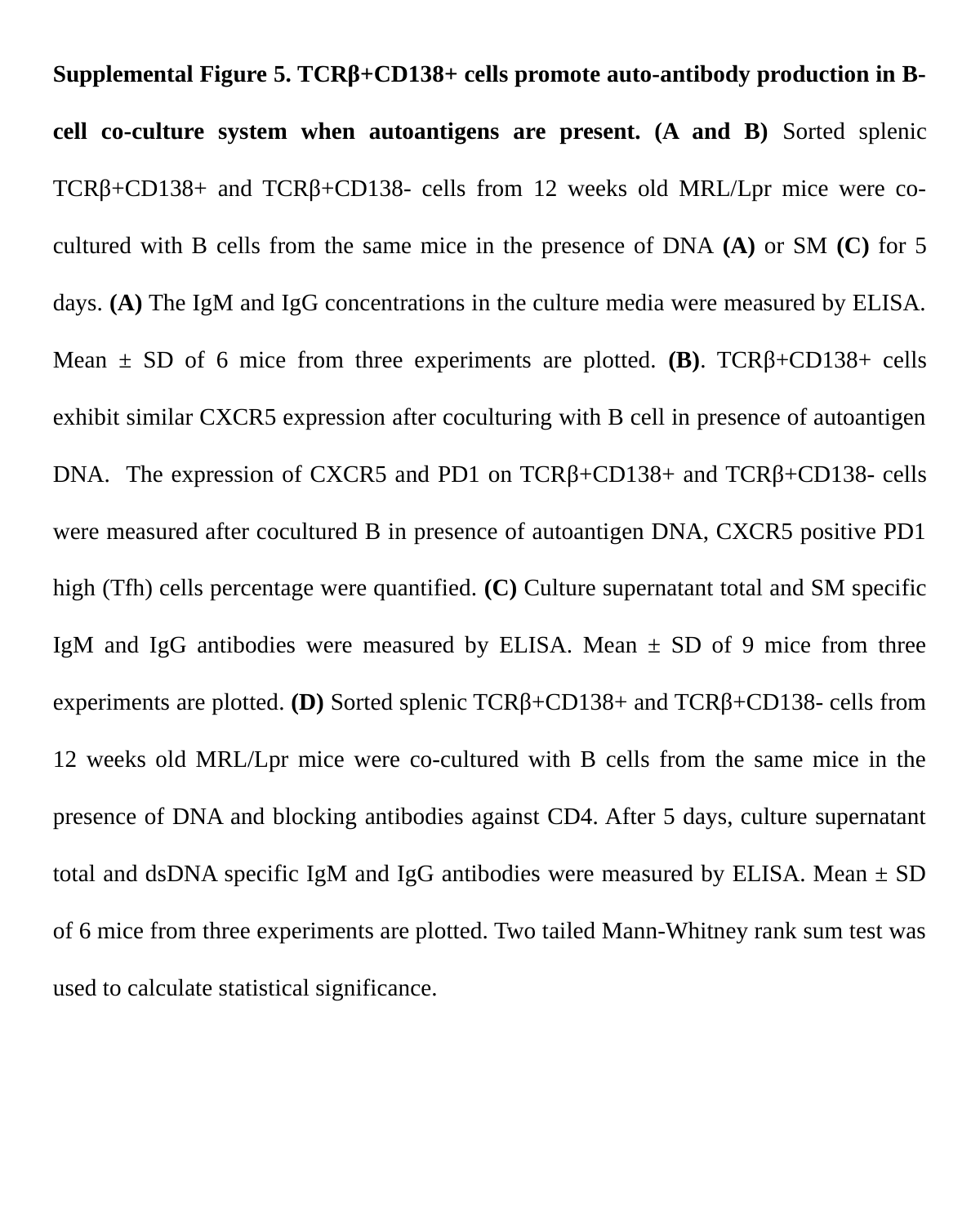

Supplemental Figure 5. TCRβ+CD138+ cells promote auto-antibody production in B-cell co-culture system when autoantigens are present. (A and B) Sorted splenic TCRβ+CD138+ and TCRβ+CD138- cells from 12 weeks old MRL/Lpr mice were co-cultured with B cells from the same mice in the presence of DNA (A) or SM (C) for 5 days. (A) The IgM and IgG concentrations in the culture media were measured by ELISA. Mean ± SD of 6 mice from three experiments are plotted. (B). TCRβ+CD138+ cells exhibit similar CXCR5 expression after coculturing with B cell in presence of autoantigen DNA. The expression of CXCR5 and PD1 on TCRβ+CD138+ and TCRβ+CD138- cells were measured after cocultured B in presence of autoantigen DNA, CXCR5 positive PD1 high (Tfh) cells percentage were quantified. (C) Culture supernatant total and SM specific IgM and IgG antibodies were measured by ELISA. Mean ± SD of 9 mice from three experiments are plotted. (D) Sorted splenic TCRβ+CD138+ and TCRβ+CD138- cells from 12 weeks old MRL/Lpr mice were co-cultured with B cells from the same mice in the presence of DNA and blocking antibodies against CD4. After 5 days, culture supernatant total and dsDNA specific IgM and IgG antibodies were measured by ELISA. Mean ± SD of 6 mice from three experiments are plotted. Two tailed Mann-Whitney rank sum test was used to calculate statistical significance.

## Slide 11
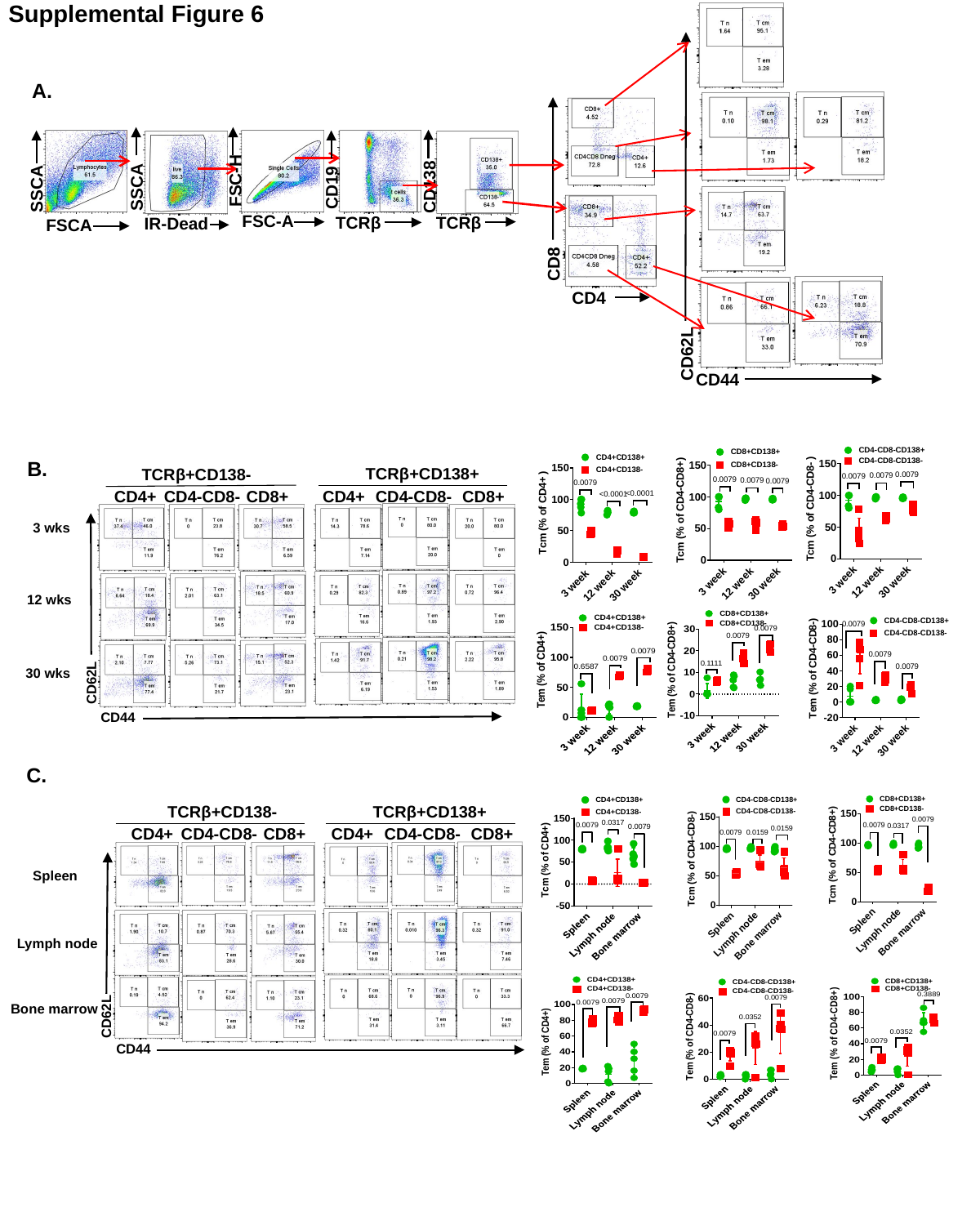

Supplemental Figure 6
A.
FSC-H
SSCA
CD138
CD19
SSCA
FSC-A
TCRβ
TCRβ
IR-Dead
FSCA
CD8
CD4
CD62L
CD44
B.
TCRβ+CD138+
TCRβ+CD138-
CD4+
CD4-CD8-
CD8+
CD4+
CD4-CD8-
CD8+
3 wks
12 wks
30 wks
CD62L
CD44
C.
TCRβ+CD138-
TCRβ+CD138+
CD4+
CD4-CD8-
CD8+
CD4+
CD4-CD8-
CD8+
Spleen
Lymph node
Bone marrow
CD62L
CD44

## Slide 12
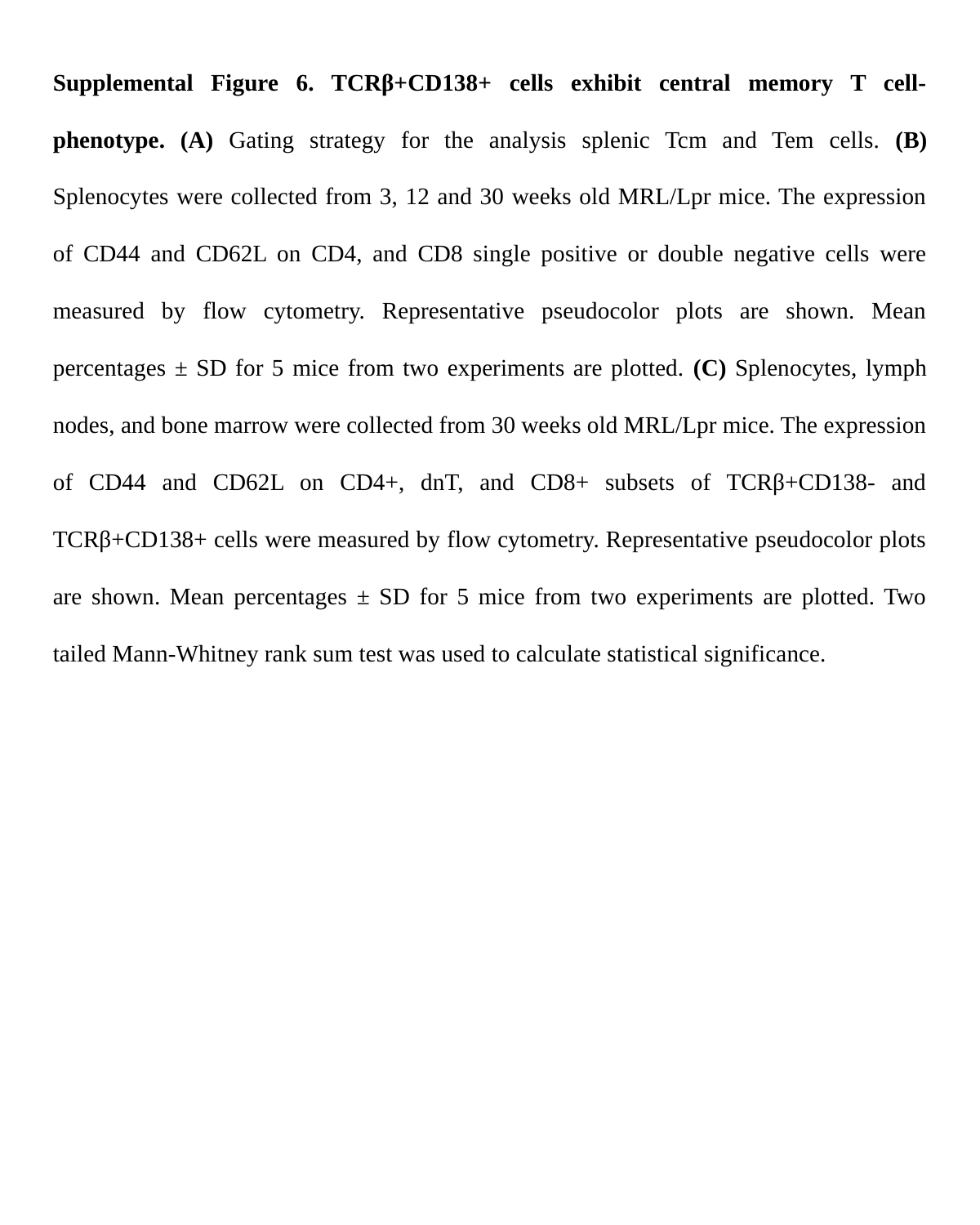

Supplemental Figure 6. TCRβ+CD138+ cells exhibit central memory T cell-phenotype. (A) Gating strategy for the analysis splenic Tcm and Tem cells. (B) Splenocytes were collected from 3, 12 and 30 weeks old MRL/Lpr mice. The expression of CD44 and CD62L on CD4, and CD8 single positive or double negative cells were measured by flow cytometry. Representative pseudocolor plots are shown. Mean percentages ± SD for 5 mice from two experiments are plotted. (C) Splenocytes, lymph nodes, and bone marrow were collected from 30 weeks old MRL/Lpr mice. The expression of CD44 and CD62L on CD4+, dnT, and CD8+ subsets of TCRβ+CD138- and TCRβ+CD138+ cells were measured by flow cytometry. Representative pseudocolor plots are shown. Mean percentages ± SD for 5 mice from two experiments are plotted. Two tailed Mann-Whitney rank sum test was used to calculate statistical significance.

## Slide 13
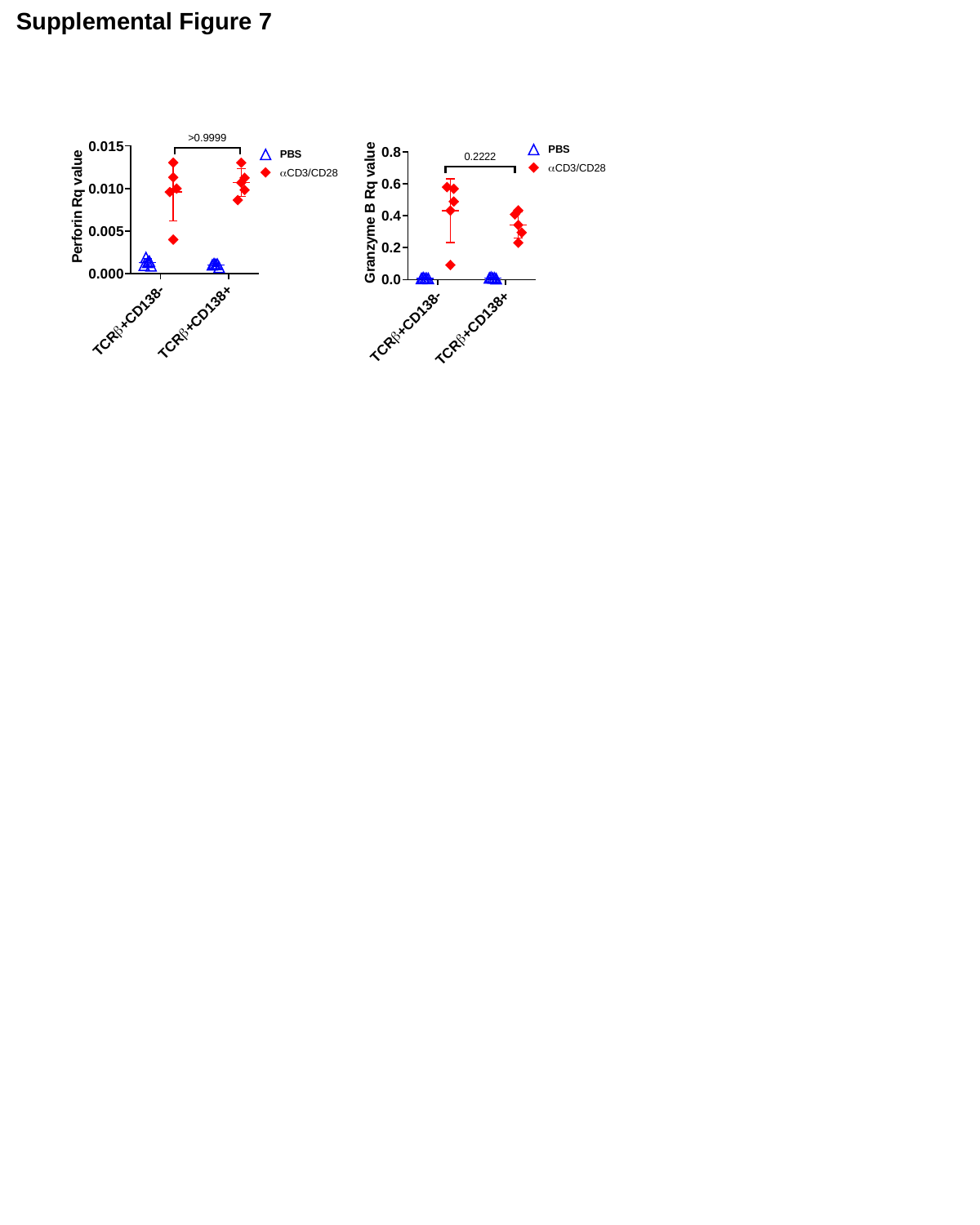

Supplemental Figure 7

## Slide 14
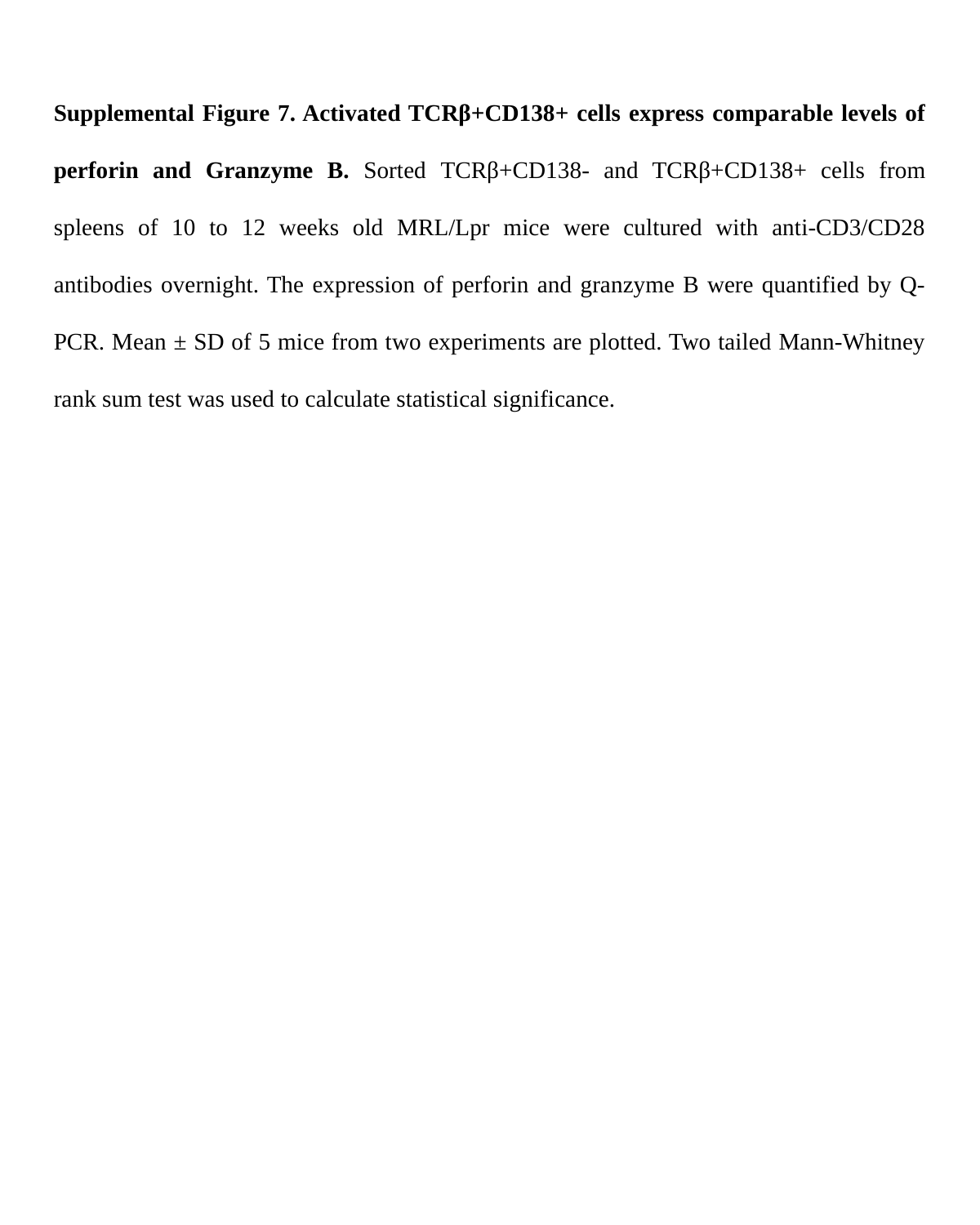

Supplemental Figure 7. Activated TCRβ+CD138+ cells express comparable levels of perforin and Granzyme B. Sorted TCRβ+CD138- and TCRβ+CD138+ cells from spleens of 10 to 12 weeks old MRL/Lpr mice were cultured with anti-CD3/CD28 antibodies overnight. The expression of perforin and granzyme B were quantified by Q-PCR. Mean ± SD of 5 mice from two experiments are plotted. Two tailed Mann-Whitney rank sum test was used to calculate statistical significance.
